# Trauma or Empowerment: Developmental Challenges and Transformative Processes Among College Students with Left-Behind Experience: A Qualitative Study

**DOI:** 10.3390/bs16071204

**Published:** 2026-07-16

**Authors:** Xiongying Li, Wanyi Zhao, Qianwen Liu, Yingjie Fan

**Affiliations:** 1 Institute of Higher Education, Lanzhou University, Tianshui South Road 222, Chengguan District, Lanzhou 730000, China; 2 School of Health and Management, Gansu University of Chinese Medicine, No. 1, Zhongyi Avenue, Heping Development Zone, Lanzhou 730101, China

**Keywords:** college students with left-behind experience, adversity-to-growth transformation, post-traumatic growth, psychological resilience, qualitative research

## Abstract

As the number of college students with left-behind experience grows, more scholars focus on their mental health and development. Most existing research highlights the negative consequences of the left-behind experience and rarely explores how individuals turn adversity into resources. In this qualitative study, semi-structured interviews were conducted with 16 college students (men: *n* = 5, women: *n* = 11; age: 20–23) who had a left-behind experience. Thematic analysis was used to examine both the negative and positive effects of the left-behind experience and the mechanisms of positive transformation. Findings suggest that left-behind experience can result in both hidden costs and cultivated resilience. Negative effects include increased emotional sensitivity, difficulty forming close relationships, and reduced feelings of belonging. Positive outcomes include greater independence, stronger responsibility, and improved social adaptability. Further analysis shows that students use three strategies—cognitive restructuring, emotional regulation, and behavioral engagement—to convert negative experiences into positive psychological resources, thereby shifting from trauma to empowerment. The study shows that left-behind experience alone does not dictate long-term outcomes. Instead, individual meaning-making and resource integration shape development. By extending resilience theory to this student group, the findings offer a strengths-based framework for psychological support and education. These findings call for university mental health services and educational policies to move beyond a deficit model and incorporate the resilience and self-regulatory strategies identified in this population.

## 1. Introduction

With rapid urbanization in China, a substantial number of rural laborers have migrated to cities, leaving behind many left-behind children. The period from 2000 to 2010 marked a critical stage during which the number of left-behind children in China grew rapidly, reaching a historical peak. During this time, many children were separated from their parents, who migrated for long-term employment, thereby creating a distinctive developmental context. As this population gradually transitions into higher education, college students with left-behind experience have attracted increasing scholarly attention. Importantly, this phenomenon is structurally embedded in China’s institutional frameworks. The *hukou* (household registration) system restricts rural migrants’ access to urban public services, forcing many parents to leave their children behind despite working in cities (Z. Liang, 2016). Regional economic disparities further drive labor migration, while limited welfare provisions for migrant families perpetuate intergenerational separation. These structural conditions shape the prevalence and nature of left-behind experiences examined in this study. It is important to clarify that the left-behind experience is conceptually distinct from clinical trauma. PTSD, as defined in the DSM-5, requires exposure to actual or threatened death, serious injury, or sexual violence—conditions that do not apply to parental separation through labor migration. The left-behind phenomenon is more accurately characterized as a chronic developmental adversity or prolonged stressor that may increase vulnerability to emotional difficulties, but it does not equate to clinical trauma. In this study, we use “trauma” in its broader, non-clinical sense to capture participants’ subjective appraisals of their experiences as painful or damaging, while maintaining conceptual clarity that the left-behind phenomenon and PTSD are distinct constructs.

Research shows that parent–child separation is both a change in family structure and a possible source of trauma. This may harm mental health (H. Liu et al., 2021). Many studies find that left-behind experience (LBE) is linked to depression, anxiety, and feeling inferior (Han et al., 2018; Yu et al., 2023).

Recently, scholars have shifted away from viewing left-behind experience solely as negative. They now focus more on resilience and growth (W. Liu et al., 2023). Many experience adversity, but not all have negative outcomes. Some students with left-behind experience show strong resilience and even post-traumatic growth (Zhao et al., 2024; Jiang et al., 2023). Left-behind experience does not always result in trauma. The effects depend on how people interpret and find meaning in it. Research points to the key role of resilience in this process (L. Liang et al., 2018). For example, students with left-behind experience sometimes have lower self-efficacy, optimism, and hope. They may also have more attachment anxiety and avoidance. Still, their resilience can be higher than that of their peers without this experience (L. Liang et al., 2020). This suggests that adversity can build adaptive abilities. Studies have found multiple factors that help this transformation. Good parent–child communication helps at home (Zhou et al., 2021). In schools, empowering educational experiences play a role (Li et al., 2025). Social support and personal gratitude also build resilience (Samoy et al., 2024). This buffers negative effects. Research confirms that resilience mediates between left-behind experience and mental health (Shi et al., 2016). Meeting basic psychological needs, like autonomy and competence, is also important for positive development (Zhao et al., 2024). Therefore, left-behind experience can be a weakness or a chance for growth. Its effect depends on both the person’s strengths and their support.

In sum, the impact of left-behind experience on college students’ mental health is characterized by a pronounced duality. Existing quantitative studies have made valuable contributions by identifying key protective and risk factors—such as psychological resilience, social support, and basic psychological need satisfaction—and clarifying their roles in shaping developmental outcomes among college students with left-behind experience. However, these studies predominantly adopt variable-centered approaches that, while informative, cannot capture the subjective, processual, and context-dependent nature of how individuals actually make sense of their left-behind experiences. What remains critically underexplored is not merely the presence or absence of certain factors, but the dynamic meaning-making processes through which these individuals actively perceive, interpret, and integrate their past adversity into their present identities and future aspirations. In particular, the mechanisms by which some students transform their left-behind experiences from potential trauma into sources of personal growth and empowerment have received little qualitative attention. To address this gap, the present study adopts a qualitative approach to examine the dual impact of left-behind experience and to explore how college students with this experience understand, cope with, and transform it over time, ultimately achieving a developmental shift from trauma to empowerment. By illuminating their lived meaning-making processes, this study contributes empirical evidence to a more nuanced understanding of the mechanisms through which left-behind experience influences development and offers implications for psychological interventions and educational support practices grounded in a strengths-based perspective.

## 2. Method

### 2.1. Participants

This study recruited university students from three public universities in China. Following Zhang’s (2006) definition of college students with left-behind experience—that is, individuals who had at least six months of left-behind experience prior to entering university—16 participants from China (men: *n* = 5, women: *n* = 11; aged 20–23) were selected using purposive sampling (Robinson, 2014). To capture variation across participants, selection considered factors such as the duration of left-behind experience and gender. Participants were coded in the chronological order of interviews. The coding format was “prefix + number,” where the prefix indicated the researcher’s initials (in pinyin) and the number was 01, 02, 03, and so forth (See Table 1). All participants provided written informed consent before data collection. Participants’ privacy and anonymity were strictly protected throughout the study, and all interviewees were assigned pseudonyms.

### 2.2. Data Collection

Interviews took place from October 2024 to December 2025. Students were interviewed at the university counseling center or in classrooms. Each interview lasted about 50–100 min. All interviews were one-on-one to protect privacy and allow open sharing. This helped us explore the issue in depth (Maxwell, 2013).

A semi-structured guide was used for interviews. The interview questions were designed based on multiple theoretical frameworks and empirical evidence. We drew on Bowlby’s attachment theory to understand participants’ emotional bonds and relationship patterns; and prior qualitative research on left-behind children in China (Sun et al., 2015), which identified key domains such as academic adjustment. Drawing on these foundations, the guide was structured to explore participants’ left-behind experiences and their current lives, including how they believe these experiences affect their well-being. Topics included academic and emotional experiences, social connections, and ways participants cope with stress. Interviews also covered self-regulation now, impacts of being left-behind, and future goals. Written informed consent was collected at the start. Each participant received RMB 200 for their time after the interview.

Data saturation was determined through an iterative process of concurrent data collection and preliminary analysis. After each interview, the research team reviewed the interview transcripts and conducted initial open coding to identify emerging themes. Recruitment continued until no new substantive themes or insights appeared in the interviews—specifically, when three consecutive interviews yielded no novel codes or thematic patterns beyond those already established in the existing dataset. At this point, we judged that the 16 participants provided sufficient information power to address our research questions. This approach aligns with the established principle that saturation is not a function of sample size per se, but rather of the richness of the data and the extent to which new data confirm or extend existing patterns (Guest et al., 2006; Hennink & Kaiser, 2022).

### 2.3. Data Analysis

All interviews were audio-recorded, transcribed verbatim, and checked by the researcher immediately after completion. Transcripts were managed and coded using NVivo 15.3.1. To examine the dual impact of left-behind experience on college students, thematic analysis was used to identify and interpret core patterns across the dataset (Braun & Clarke, 2006, 2021). The analytic process involved: (1) familiarization with the data; (2) generating initial codes; (3) collating codes into potential themes; (4) reviewing themes against coded extracts and the full dataset; (5) defining and naming themes; (6) producing the report.

After becoming familiar with the data, the researcher conducted inductive semantic coding. Codes were then integrated based on conceptual similarity to generate higher-order potential themes. Through iterative discussion and refinement, core themes that cut across the dataset were ultimately identified. To enhance the accuracy and credibility of the final coding, participant feedback was solicited to confirm that the researcher’s interpretations were consistent with participants’ perspectives (Creswell & Miller, 2000).

To further ensure the rigor of this qualitative study, we adopted Erlandson’s (1993) trustworthiness framework. Credibility was strengthened through member checking, in which participants reviewed and confirmed the accuracy of the thematic interpretations, as well as through peer debriefing with two independent researchers who reviewed the coding process and challenged potential biases. Dependability was addressed by maintaining a comprehensive audit trail that documented all analytic decisions—from initial coding to theme refinement—and by conducting a code-recode procedure in which the first author revisited the same transcripts after a two-week interval to assess coding consistency. These strategies collectively enhance the methodological trustworthiness of the study.

## 3. Results

This study sought to examine, through thematic analysis of interview data, both the positive and negative impacts of left-behind experience on college students and the strategies through which they transform adverse influences into constructive action. The findings are presented in two parts. The first identifies two overarching themes concerning the multidimensional effects of left-behind experience. The second delineates the strategies by which college students with left-behind experience actively convert negative influences into positive developmental outcomes.

### 3.1. The Multidimensional Impact of Left-Behind Experience

The multidimensional impact coding results for left-behind experiences are shown in Table 2.

#### 3.1.1. Positive Impact: Cultivated Resilience

(1) Independence and Autonomy

Extended exposure to a left-behind environment fostered early self-reliance at both practical and psychological levels. In the absence of sustained parental presence, many participants were required to manage daily responsibilities and navigate emotional challenges independently, demonstrating pronounced autonomy in decision-making, problem-solving, and self-regulation. This independence was evident not only in personal life but also in interpersonal contexts, where participants frequently assumed leadership or caregiving roles within peer and intimate relationships.

“My personality has become more independent. I can accomplish many things on my own without needing others’ help. I have my own way of thinking, and I consider things more thoroughly.”

(KLY-03)

“Through my left-behind experience, I developed independence and autonomy, and formed relatively strong psychological resilience and adaptability. These qualities will benefit my future social adaptation.”

(WYY-07)

“I have been quite independent since childhood. In daily life, I took on many responsibilities myself. Emotionally, I had to work through things on my own. In peer relationships, I often take on a guiding role. In intimate relationships, I also tend to care for others.”

(WYY-16)

(2) Tenacity and Sense of Purpose

Growing up with limited direct parental support and care, some college students with left-behind experience gradually internalized a strong drive for achievement and resilience. When confronted with intergenerational value conflicts or constrained family resources, they often oriented their behavior around clearly defined personal goals. In pursuing these goals, they cultivated sustained perseverance and a strengthened capacity to cope with setbacks.

“My grandparents were born in the 1940s and 1950s, so their thinking is relatively conservative. There were many issues, such as a preference for sons over daughters. This made me very strong-willed. Since childhood, I have told myself that only by becoming truly capable could I go far away and do what I want to do.”

(CSH-01)

“My personality and values are mainly reflected in my tenacity and persistence. I do not give up on things easily. Once I make a decision, I will see it through to the end.”

(YJS-04)

(3) Responsibility and Empathy

Early awareness of family circumstances led many college students with left-behind experience to recognize and assume family responsibilities at a relatively young age. They often took on practical and emotional roles within the household in the absence of parental presence. At the same time, their own experiences of relational deprivation heightened their sensitivity to others’ hardships and strengthened their empathic concern. As a result, they demonstrated a pronounced prosocial orientation, frequently engaging in volunteer service or offering support in everyday interactions, reflecting a strong sense of social responsibility and empathy.

“As I grew older, I tried to help people who needed support. After entering university, I participated in many volunteer activities. I truly enjoy helping others. When I see them, I see people in need, and at the same time, I see my younger self. In college, I joined an association that supports disadvantaged children. We helped left-behind children with their homework and played with them.”

(YJS-04)

“Sometimes I place great importance on my responsibility to my family. When my parents are away, I can take on some of the major family matters so my grandparents do not have to worry as much. I think this has made me more responsible and considerate.”

(XMH-08)

“I feel that I have a strong sense of responsibility. My family has faced many difficulties, which motivates me to study harder and change our family’s situation.”

(NC-14)

(4) Social Perceptiveness and Adaptability

Navigating complex and shifting interpersonal environments required college students with left-behind experience to become attuned to others’ emotions and intentions. Over time, they developed heightened social perceptiveness and interpersonal adaptability, enabling them to detect subtle social cues, initiate relationships proactively, and adjust flexibly across diverse social contexts.

“I may be more likely to notice when others need help or someone to talk to. I can also sense whether someone is being kind or impatient when speaking with me. This may be related to how I learned to read social cues when I was young.”

(CSH-01)

“I am good at reading social cues and very attentive to others’ feelings. I can usually take good care of people around me.”

(LMY-06)

“I am more proactive and outgoing in making friends. I maintain relationships with sincerity and friendliness.”

(XM-09)

“I think I am relatively outgoing and more skilled at interacting with others and making friends.”

(LSN-12)

#### 3.1.2. Negative Impact: Latent Costs

(1) Heightened Emotional Sensitivity and Low Self-Esteem

Prolonged emotional absence and insufficient parental presence undermined some participants’ sense of self-worth, manifesting in heightened sensitivity to others’ evaluations and persistent self-doubt. In interpersonal interactions, they tended to overinterpret social cues, experience feelings of inferiority, and, under perceived social pressure, suppress their own needs in order to accommodate others.

“Since entering university, I have increasingly realized that I struggle with low self-esteem and sensitivity. Sometimes I unconsciously go along with others. For example, when everyone goes out to dinner together, I may not want to go, but to avoid embarrassment, I still choose to compromise. There are many similar situations.”

(LCY-02)

“I tend to be highly sensitive and very good at reading social cues. I can tell whether someone feels happy or unhappy after I say something, but this often makes me feel distressed. I may overthink a subtle expression from others. Even if it is unconscious and not directed at me, I interpret it as a negative evaluation of myself.”

(ZQ-13)

(2) Difficulties in Intimate Relationships

Early disruptions in attachment relationships undermined participants’ confidence and their capacity to establish secure, stable intimate relationships in adulthood. Many reported avoidance, fear, or insecurity within close relationships, difficulty expressing emotions openly, or a tendency toward excessive control.

“I have never been very good at establishing and maintaining intimate relationships. In popular terms, you could say I am somewhat ‘incapable of love.’”

(ZSR-10)

“When I was young, I was separated from my parents. Even now, I still feel insecure. I am afraid of love and of others abandoning me. If I love someone, I do not dare to express it openly. I worry that they might think I am not good enough, or that, like my parents, they might leave me. Since childhood, I have always carried this sense of insecurity.”

(KJ-15)

(3) Emotional Distance and Ambivalence in Parent–Child Relationships

Prolonged separation weakened emotional bonds between college students with left-behind experience and their parents. Even after later reunification, substantial communication barriers and emotional distance often persisted. Participants described ambivalent feelings toward their parents—simultaneous understanding and conflict—marked by alienation and resentment, alongside attempts at closeness that were hindered by enduring psychological barriers.

“My relationship with my parents is not very good. Because we have had different life experiences, we have few shared topics. I have tried to get closer to them, but there is always a gap—both psychologically and in communication.”

(CSH-01)

“There are many things I am unwilling to tell my parents. I feel closer to my siblings. It may be due to the generation gap or because they were absent for so long, but there is still some distance between us.”

(LCY-02)

“I still carry deep emotional wounds. I feel that the reason I am not very close to my parents, even now, is that I believe they did not treat me well when I was young.”

(KJ-15)

(4) Emotional Suppression

College students with left-behind experience tended to cope with emotional distress independently, often developing a pattern of emotional suppression and reluctance to seek external support. Although this strategy helped them maintain outward stability in the short term, it may have contributed to longer-term emotional depletion and psychological exhaustion.

“In some situations, I do not know what I should do. I feel uncomfortable asking others for help, so many things have to be handled on my own. This leaves me feeling emotionally depleted. When I am unhappy, I do not tell others because I am afraid of burdening them.”

(KLY-03)

“When I have worries, I usually do not show them in front of my mother. I deal with them by myself. I may feel emotionally low for a few days, and then it passes. If I really cannot cope, I try to distract myself in other ways.”

(WYY-07)

(5) Diminished Sense of Belonging

Frequent residential transitions during childhood, or prolonged residence in boarding schools and other collective settings, deprived college students with left-behind experience of stable and continuous sources of emotional security. As a result, many described a persistent sense of rootlessness and a weakened sense of belonging within their families.

“I was always experiencing changes in environment—going from school to home each week, then to my grandmother’s house or my maternal grandmother’s house. Moving between these places made the world feel unstable, as if there was no safe place that truly belonged to me. I always had this sense of drifting.”

(ZQ-13)

“I often feel displaced, as if I do not have a stable sense of belonging anywhere.”

(KJ-15)

(6) Challenges in Self-Regulation and Excessive Engagement in Entertainment

The relative lack of external supervision and guidance in early life created challenges for self-regulation among college students with left-behind experience. In particular, some reported difficulty maintaining a balance between academic responsibilities and leisure activities, occasionally leading to excessive engagement in entertainment behaviors.

“From the first grade of primary school, I was a child who did not complete homework and ranked near the bottom of the class. Once, after winter vacation, I had not finished my homework and was asked by the teacher to stand at the front of the classroom with two other boys who also ranked at the bottom. In fifth grade, because I stayed up until one or two in the morning watching TV dramas, my father decided to relocate me to the city where he worked.”

(LXP-05)

“I remember that during a period in middle school, I became very lax about studying and excessively engaged in watching TV dramas and playing video games. The teacher strongly pointed out that my grades had declined significantly.”

(XMH-08)

### 3.2. Proactive Transformation Strategies

The coding results of proactive transformation strategies among left-behind college students are shown in Table 3.

#### 3.2.1. Cognitive Restructuring: Meaning-Making

(1) Acceptance, Gratitude, and Perspective Transformation

When confronting the emotional absence and psychological challenges associated with left-behind experience, college students engaged in active cognitive restructuring, reframing earlier deprivation as a formative developmental resource. Many sought to understand their parents’ decisions or expressed gratitude toward caregivers during the left-behind period, thereby facilitating emotional reconciliation with their families and integrating this experience into a coherent and growth-oriented self-narrative.

“I have used my high sensitivity to accomplish small things that make me proud and to become the person I wanted to be. Even now, I remain grateful for this experience. In the end, I believe it depends on one’s inner drive.”

(CSH-01)

“I have tried to communicate more with my parents, and my mother often talks with me as well. I now treat her like a friend. Whenever I share my thoughts and decisions, she listens, supports, and respects my views. Although I no longer rely on their care in the same way, I understand more clearly that everyone needs love and companionship. Therefore, one of my core values is that family presence is essential.”

(YJS-04)

“After entering university, I realized that family is the foundation for establishing oneself in society, so my relationship with my family gradually improved. I can now overlook, or rationally respond to, generational differences between my parents and young people today.”

(LXP-05)

(2) Attentional Shift and Future Orientation

To avoid becoming preoccupied with past adverse experiences, some participants deliberately redirected their focus toward present goals and future aspirations. By setting clear life objectives and investing psychological energy in domains within their control, they mitigated the negative impact of early experiences while strengthening their sense of agency and hope.

“I hope my future workplace will be close to home so that I can visit my grandparents often. I may become a busy nurse, or perhaps change careers, as long as I can secure a stable income.”

(LMY-06)

“Because there were certain gaps in my childhood, I hope that if I have children in the future, they will not experience the same kind of absence. I want a stable job not too far from home, a happy family, and a small house in our county—living a life that I consider truly fulfilling.”

(XMH-08)

(3) Seeking Meaning and Establishing Value Anchors

In the absence of consistent emotional support, college students with left-behind experience actively explored and constructed personal value systems through reading, dialogue, and community engagement. Many transformed their lived experiences into motivation for prosocial action, or consolidated core beliefs—such as personal worth and social responsibility—as enduring value anchors that provided direction and purpose.

“I would like to engage in community-level work and see children like my younger self. I have become more compassionate and tend to resolve problems more calmly. I pursue a stable life and hope to live peacefully. If I have the ability, I would like to help left-behind children by offering them emotional support and using my own experiences to assist them, so that every child like the former me can grow up healthy and happy.”

(KLY-03)

“I think the greatest impact of left-behind experience on me is that I must strive for everything on my own; I cannot rely on others. I have to ensure my own value in order to establish myself. I also believe that only by possessing and creating value can I obtain a stable and well-paid position.”

(LCY-02)

#### 3.2.2. Emotional Regulation: Developing Support Pathways

(1) Seeking Alternative Emotional Support

When their families were unable to provide sufficient emotional responsiveness, participants developed the capacity to seek support from alternative relational networks. They extended emotional reliance to siblings, extended family members, peers, and teachers, deriving understanding and companionship from these substitute attachments. Such alternative bonds partially compensated for the limitations of early parent–child interaction.

“My maternal grandparents, my aunt, my older sister, and my cousins all played indispensable roles in my life. My aunt has always been like a second mother to me. When I was young, she gave me the feeling of having a mother.”

(YJS-04)

“When I was young, I had a close relationship with my grandparents. It felt like whether my father was around or not made much difference. He did not influence me that much. My grandfather, especially, supported me more in daily life.”

(DDB-11)

(2) Expressive Activities and Reflective Self-Dialogue

When confronted with difficult-to-articulate emotional experiences, college students with left-behind experience engaged in expressive and reflective practices—such as writing, listening to music, reading, and other creative activities—as means of emotional processing and self-dialogue. These strategies provided a safe emotional outlet, facilitated self-awareness, and supported emotion regulation.

“I listen to music and give myself more time and space, trusting that I can do better.”

(KLY-03)

“I cultivate long-term interests such as reading, public speaking, and photography. By developing both internal qualities and external skills, I explore my interests and meet diverse people. Through self-improvement, I gradually become better.”

(YJS-04)

“I take deep breaths, make plans, and listen to music—basically, I try to resolve difficulties on my own.”

(ZSR-10)

(3) Seeking Professional Psychological Support

With increased awareness of mental health, college students with left-behind experience also reported proactively seeking psychological counseling or clinical assessment. Through professional support, they were able to identify emotional difficulties and receive guidance for cognitive and behavioral adjustment.

“Eventually, I sought psychological counseling. The counselor said I had an anxious attachment style. In romantic relationships, I would feel insecure, experience a strong need for control, be overly sensitive, and struggle with distrust that could feel suffocating. I tended to want to withdraw passively, and breakups seemed inevitable. The counselor advised me to change my patterns of thinking and behavior and to build core self-confidence.”

(YJS-04)

“During university, a period of psychological counseling helped me accept myself more fully. Later, I completed assessments for autism as well as anxiety and depression, and the results suggested that I was outside the typical range. I subsequently sought consultation twice at a tertiary-level psychiatric hospital. The diagnosis included sensory integration difficulties and challenges in intimate relationships. The doctor recommended swimming and regular counseling. I now swim once a week and continue my studies with a positive mindset, which has been very helpful in maintaining both my physical health and emotional well-being.”

(LXP-05)

#### 3.2.3. Behavioral Engagement: Capacity Building

(1) Self-Regulated Academic Motivation

For many college students with left-behind experience, academic achievement was perceived as a critical pathway for improving both their own and their families’ life trajectories. Participants frequently demonstrated strong self-regulated learning and intrinsic motivation, deliberately minimizing environmental distractions to sustain academic engagement.

“Repeating the high school entrance examination had the greatest impact on me. During my three years of high school, I strictly limited my mobile phone use. To avoid distractions, I chose to live on campus and even stayed at school on weekends. At one point, I remained at school for an entire month without going home.”

(XM-09)

“My academic performance has not been significantly better than others; I have generally been at an upper-middle level. However, my intrinsic drive is relatively strong. I often experience anxiety, but now I am learning to channel it into productive motivation rather than staying anxious all day and feeling unable to act as before.”

(WYY-16)

(2) Economic Autonomy and Family Responsibility

Economic independence was widely regarded as a concrete expression of personal autonomy. Many participants hoped to alleviate their families’ financial burdens, assume familial responsibilities, and derive a sense of security through financial self-sufficiency.

“I strongly hope that I will develop sufficient capacity to contribute to my family’s well-being.”

(WYY-07)

“I want to be independent and not cause my parents to worry too much. When I want to buy something, I prefer to use money that I have earned or saved myself. With extra money, I bought each of my parents a pair of shoes and prepared Lunar New Year gifts for my parents, grandparents, and maternal grandmother.”

(DDB-11)

“When I think about giving back, it is mainly toward my grandparents. I truly feel that they have endured many hardships.”

(ZQ-13)

(3) Intentional Social Engagement and Skill Development

Although some college students with left-behind experience had earlier adverse interpersonal experiences, several participants deliberately engaged in social skills development to overcome social anxiety, expand their interpersonal networks, and treat social interaction as a developmental resource.

“We need to learn to listen to others’ opinions in order to go further. Positive interpersonal relationships help us understand many things, improve our mood, and increase motivation for other tasks. Therefore, it is important to value the influence of relationships.”

(KLY-03)

“I think it was because I left my original environment. When I first entered university, everything felt new. I began consciously to make breakthroughs—to try things I had never done before. I was afraid of meeting strangers, so I made myself meet one new person every day. I practiced public speaking and impromptu speeches, met diverse people, and gradually overcame social anxiety.”

(YJS-04)

## 4. Discussion

Drawing on qualitative interviews, this study examined the dual impact of left-behind experience on college students’ development and the proactive strategies they use to transform adversity. The findings suggest that left-behind experience should not be understood as a singular traumatic event, but rather as a complex developmental process encompassing both deprivation and potential, setback and growth. By illuminating cognitive, emotional, and behavioral dimensions of this process, the study delineates a dynamic pathway from trauma to empowerment and extends theoretical perspectives on adversity and resilience.

Consistent with prior research (W. Liu et al., 2023), the present findings confirm the dual nature of left-behind experience. Negative influences were primarily reflected in heightened emotional sensitivity and low self-esteem, difficulties in intimate relationships, and a diminished sense of belonging, echoing earlier evidence of comparatively lower levels of social trust (Yang et al., 2023). At the same time, positive outcomes included enhanced independence, resilience, responsibility, and social perceptiveness, suggesting that individuals may develop distinctive psychosocial competencies through compensatory and adaptive processes in response to adversity (Zhao et al., 2024; Jiang et al., 2023). These results underscore that left-behind experience itself does not predetermine developmental trajectories; rather, outcomes depend on how individuals subsequently interpret, integrate, and assign meaning to this formative experience. However, beyond simply confirming these dual patterns, our findings offer three further interpretive insights that extend the current literature. First, the coexistence of negative and positive outcomes within the same individuals suggests that these are not independent pathways—as often implied in variable-centered quantitative studies—but rather mutually constitutive dimensions of a single developmental process. The same experiences of separation that generate emotional insecurity simultaneously create conditions for heightened self-reliance, suggesting that adversity does not simply “add” both costs and benefits but fundamentally restructures the individual’s developmental landscape in ways that render costs and benefits inseparable. Second, our data reveal that the weighting between adversity and growth is not static but dynamically shifts across developmental stages and life contexts. Participants in our study often described how the same memory could be reinterpreted differently over time—what felt like abandonment in early adolescence later came to be reframed as an opportunity for independence, and vice versa. This temporal fluidity challenges cross-sectional accounts of left-behind effects and points to the crucial role of retrospective narrative construction in shaping long-term developmental outcomes. Third, these findings collectively suggest that the left-behind experience functions less as a fixed “risk factor” and more as a developmental contingency—its impact is fundamentally contingent upon the interpretive frameworks, social resources, and life opportunities available to individuals as they navigate their post-left-behind lives. This contingency perspective moves beyond the deficit-versus-strength debate to reframe the left-behind experience as a biographically significant event whose meaning and consequences are actively negotiated over time, rather than passively endured or deterministically suffered.

Moreover, the findings demonstrate that individuals transform negative experiences into constructive resources through proactive strategies at three interrelated levels: cognitive restructuring, emotional regulation, and behavioral engagement. Cognitive restructuring was reflected in the reinterpretation and meaning-making of left-behind experience, consistent with the central role of meaning construction in post-traumatic growth theory (Tedeschi & Calhoun, 2004). Emotional regulation strategies revealed that, in the absence of sufficient familial emotional support, individuals cultivated diverse channels for emotional expression and support-seeking, thereby broadening existing understandings of social support sources (Zhou et al., 2021). At the behavioral level, participants translated internal motivation into tangible competencies—through sustained academic engagement, economic autonomy, and intentional social participation—demonstrating strong self-regulation and goal-directed action. These findings further support the central role of basic psychological need satisfaction in promoting adaptive development (Adams et al., 2017). Importantly, the transformation process was not solely intrapsychic but emerged from the dynamic interaction between internal resources and environmental conditions. The quality of family communication (Zhou et al., 2021), empowering experiences within school contexts (Li et al., 2025), and broader social support systems all provided essential resources for positive adaptation. This aligns with the systemic perspective of resilience theory, which conceptualizes resilience not merely as an individual trait but as a capacity constructed through ongoing person–environment interaction (Masten, 2001). This systemic understanding is particularly pertinent to the left-behind population, whose developmental environment is characterized by both structural deprivation and potential resources. Our findings suggest that the resilience exhibited by these students does not simply reflect individual “toughness,” but emerges from their active identification and mobilization of available environmental supports—a process that itself is mediated by their cognitive interpretations of their circumstances. This reframes resilience not as a personal attribute to be measured, but as a dynamic, relational process that educational and family interventions can actively cultivate by strengthening environmental resources and empowering students’ meaning-making capacities.

This empirical study provides several important contributions to both theory and practice. At the theoretical level, the findings underscore the pivotal role of person–environment interaction in transforming left-behind experience, aligning with the systemic perspective of resilience theory (Masten, 2001). The development of psychological resilience depends not only on individuals’ cognitive and emotional regulatory capacities but also on supportive responses from external systems, including family, school, and broader society. Accordingly, the long-term outcomes of left-behind experience emerge from the ongoing co-construction of personal psychological resources and socio-contextual resources. Unlike most prior research that has predominantly employed variable-centered quantitative designs to identify correlates of left-behind status, the present study offers a process-oriented qualitative account. Our unique contribution lies in two aspects. First, we explicitly connect resilience theory, post-traumatic growth, and meaning-making into a unified analytical framework, demonstrating that the transition from trauma to empowerment is not merely a reflection of resilient traits but is actively constructed through dynamic cognitive reappraisal and narrative integration over time—a process that has been largely black-boxed in existing quantitative literature. Second, rather than treating negative and positive outcomes as separate dimensions to be correlated with distinct predictors, we show that they are deeply intertwined and mutually constitutive, emerging from the same adaptive response to prolonged family separation. This integrative perspective challenges the prevailing dichotomous framing in the literature and offers a more ecologically valid account of how adversity shapes development. Collectively, these contributions advance a more nuanced, person-centered theoretical understanding of left-behind experience that moves beyond the deficit-versus-strength dichotomy toward a process-oriented and meaning-centered developmental framework. At the practical level, the study advocates for a more developmentally oriented and integrative support framework. For college students with left-behind experience, educational and social interventions should move beyond deficit-compensation models and adopt strengths-based approaches that emphasize resource activation and sustained developmental support. Specifically, families should enhance communication quality and emotional responsiveness; schools should foster inclusive and empowering educational environments; and communities and social institutions should establish coordinated networks that integrate formal and informal sources of support. Through such collaborative efforts, it is possible to facilitate a shift from adaptation focused primarily on survival to growth-oriented developmental flourishing.

Families, schools, communities, and mental health professionals each play critical roles in fostering the positive development of college students with left-behind experience. Based on the present findings, three practice-oriented recommendations are proposed. First, a developmentally sequenced support system should be established. During earlier stages, coordinated efforts among families, schools, and communities are needed to provide stable emotional care and behavioral guidance for left-behind children, thereby mitigating developmental risks associated with prolonged emotional neglect. At the university level, mental health education, career guidance, and group-based interventions can help students identify personal strengths, reconstruct growth narratives, and enhance psychological capital. Second, strength-based intervention approaches should be advanced. Practitioners should avoid pathologizing left-behind experience and instead recognize and reinforce the resilience, responsibility, and adaptability that individuals have already demonstrated. Approaches such as narrative therapy may strengthen positive self-identity and facilitate the integration of traumatic experiences into a coherent developmental narrative. Third, universities should cultivate diversified support networks, including peer support groups, mentorship programs, and financial assistance mechanisms tailored to students with left-behind experience. By providing emotional support, role modeling, and material resources, such initiatives can alleviate a diminished sense of belonging and economic strain, thereby creating a more inclusive environment conducive to competence development and long-term well-being. Fourth, these efforts must be accompanied by structural policy reforms that address the root causes of left-behind experiences. Governments should prioritize improving rural education and welfare, and investing in local economic development to reduce the necessity of family separation.

Several limitations should be acknowledged. First, the sample was drawn from three universities; future research could expand the sampling frame to include students from diverse regions and institutional types to enable comparative analysis. Furthermore, given the cross-sectional design, the present study was unable to capture the temporal dynamics of the transformation process. Subsequent research employing longitudinal designs or life-history interviews may further elucidate critical turning points and underlying mechanisms in the trajectory from trauma to empowerment. Third, the findings rely on retrospective self-reports, which may be subject to recall bias and social desirability. Future studies could incorporate multiple data sources to triangulate findings. Fourth, this study was conducted within China’s specific socio-cultural context, where left-behind experiences are closely tied to the *hukou* system and rural-urban migration. The transferability of our findings to other cultural settings may therefore be limited, and cross-cultural research is needed to test their broader applicability.

## 5. Conclusions

By closely examining the lived experiences and transformation strategies of college students with left-behind experience, this study provides qualitative evidence illuminating the complex relationship between adversity and growth. Left-behind experience may represent a latent psychological cost, yet it can also function as a context for cultivating resilience. Its developmental trajectory is shaped by ongoing interactions between individuals and their environments. The growth narratives of college students with left-behind experience exemplify how meaning can be constructed amid deprivation, revealing an important developmental pathway from adversity to empowerment. These findings contribute to strengths-based approaches by demonstrating that left-behind students actively reframe adversity using cognitive, emotional, and behavioral strategies. Rather than focusing on deficits, interventions should identify and amplify these existing adaptive capacities. The meaning-making processes we documented—cognitive reappraisal and narrative integration—offer concrete entry points for strengths-based practices that empower students. This study thus provides an empirical foundation for mental health and education policies that recognize left-behind experiences as potential assets to be harnessed, not merely problems to be remedied.

## Figures and Tables

**Table 1:** Demographic Characteristics of Participants.

Participant ID	Duration of Left-Behind Experience	Gender	Age
CSH-01	12 years	woman	22
LCY-02	14 years	man	21
KLY-03	3 years	man	21
YJS-04	14 years	man	23
LXP-05	10 years	woman	23
LMY-06	15 years	woman	20
WYY-07	11 years	woman	22
XMH-08	9 years	woman	23
XM-09	6 years	woman	20
ZSR-10	3 years	woman	21
DDB-11	11 years	woman	23
LSN-12	5 years	man	21
ZQ-13	12 years	woman	23
NC-14	3 years	man	21
KJ-15	13 years	woman	23
WYY-16	8 years	woman	23

**Table 2:** The Multidimensional Impact of Left-Behind Experience.

Theme	Subtheme	Definition
Positive Impact: Cultivated Resilience	Independence and Autonomy	A strong tendency toward self-reliance in life decisions, problem-solving, and psychological adjustment, with limited dependence on others.
	Tenacity and Sense of Purpose	The development of robust psychological resilience, perseverance, and a clearly defined achievement drive in the face of adversity.
	Responsibility and Empathy	Early recognition of family circumstances and proactive assumption of familial responsibilities; heightened empathic sensitivity to others’ suffering rooted in personal experience.
	Social Perceptiveness and Adaptability	Enhanced ability to interpret social cues and to establish initial interpersonal connections efficiently within complex social environments.
Negative Impact: Latent Costs	Heightened Emotional Sensitivity and Low Self-Esteem	Increased sensitivity to interpersonal evaluation and perceived insufficiency of familial affection, accompanied by a persistently diminished sense of self-worth.
	Difficulties in Intimate Relationships	Challenges in establishing and maintaining deep, secure intimate relationships, manifested in avoidance, fear, excessive compliance, or controlling tendencies.
	Emotional Distance and Conflict in Parent–Child Relationships	Emotional barriers, communication difficulties, or ambivalent and sometimes resentful relationships with parents.
	Emotional Suppression	A tendency to internalize negative emotions and refrain from seeking familial support, resulting in an emotionally avoidant coping pattern.
	Diminished Sense of Belonging	A persistent sense of rootlessness associated with frequent residential transitions or prolonged boarding experiences.
	Challenges in Self-Regulation and Excessive Engagement	Difficulties in self-control, particularly in relation to online or entertainment activities, stemming from limited early external supervision and guidance.

**Table 3:** Proactive Coping Strategies for Transformation.

Theme	Subtheme	Definition
Cognitive Restructuring: Meaning-Making	Acceptance, Gratitude, and Perspective Transformation	Reframing left-behind experience as a valuable resource in shaping one’s current self, or expressing gratitude toward caregivers.
	Attentional Shift and Future-Oriented Focus	Reducing the impact of past pain by directing focus toward the present and future, where personal effort can lead to meaningful change.
	Seeking Meaning and Value Anchors	Actively constructing and revising one’s personal values and beliefs through social networks and interactions with more experienced individuals to combat instability.
Emotional Adjustment: Developing Support Channels	Turning to Alternative Sources of Emotional Support	Seeking emotional comfort and understanding from friends, partners, teachers, or other relatives.
	Expressive Writing and Artistic Expression	Engaging in emotional expression and self-dialogue through journaling, music, literature, and other creative outlets.
	Seeking Professional Psychological Help	Proactively seeking psychological counseling or diagnosis to address deep-rooted psychological issues through evidence-based methods.
Behavioral Engagement: Expanding Competencies	Self-Driven Academic Engagement	Viewing learning as a core pathway for altering life circumstances, and dedicating significant effort to academic achievement.
	Economic Autonomy and Responsibility	A strong desire for financial independence, saving money to reduce family burdens, demonstrate one’s value, and gain a sense of security.
	Deliberate Social Engagement and Social Skill Development	Actively participating in clubs, activities, and practicing social skills to overcome interpersonal fears and expand social networks.

## Data Availability

The original contributions presented in this study are included in the article. Further inquiries can be directed to the corresponding authors.
